# Chronic unpredictive mild stress leads to altered hepatic metabolic profile and gene expression

**DOI:** 10.1038/srep23441

**Published:** 2016-03-23

**Authors:** Hong-mei Jia, Qi Li, Chao Zhou, Meng Yu, Yong Yang, Hong-wu Zhang, Gang Ding, Hai Shang, Zhong-mei Zou

**Affiliations:** 1 Institute of Medicinal Plant Development, Chinese Academy of Medical Sciences and Peking Union Medical College, No. 151 Malianwa North Road, Haidian District, Beijing 100193, China

## Abstract

Depression is a complex disease characterized by a series of pathological changes. Research on depression is mainly focused on the changes in brain, but not on liver. Therefore, we initially explored the metabolic profiles of hepatic extracts from rats treated with chronic unpredictive mild stress (CUMS) by UPLC-Q-TOF/MS. Using multivariate statistical analysis, a total of 26 altered metabolites distinguishing CUMS-induced depression from normal control were identified. Using two-stage receiver operating characteristic (ROC) analysis, 18 metabolites were recognized as potential biomarkers related to CUMS-induced depression via 12 metabolic pathways. Subsequently, we detected the mRNA expressions levels of apoptosis-associated genes such as *Bax* and *Bcl-2* and four key enzymes including *Pla2g15, Pnpla6, Baat* and *Gad1* involved in phospholipid and primary bile acid biosynthesis in liver tissues of CUMS rats by real-time qRT-PCR assay. The expression levels of *Bax, Bcl-2, Pla2g15, Pnpla6* and *Gad1* mRNA were 1.43,1.68, 1.74, 1.67 and 1.42-fold higher, and those of *Baat, Bax/Bcl-2* ratio mRNA were 0.83, 0.85-fold lower in CUMS rats compared with normal control. Results of liver-targeted metabonomics and mRNA expression demonstrated that CUMS-induced depression leads to variations in hepatic metabolic profile and gene expression, and ultimately results in liver injury.

Depression is a widespread psychiatric illness ranked by the World Health Organization (WHO) as one of the most burdensome diseases of society^1^,^2^. It is characterized by a series of pathological stages associated with stressful events, leading to low morale, weight loss and anhedonia^3^,^4^. Most of the reports on depression mainly focus on changes in the brain areas including frontal cortex, hippocampus and striatum^5^,^6^. However, no reports are available on the effect of depression on other organs excepted heart^7^.

Liver is a center of substrate and energy metabolism. It plays diverse biological roles and impacts other systems of the body. Patients afflicted with chronic liver disease^8^, acute toxic hepatic injury^9^, non alcoholic fatty liver^10^ and liver cirrhosis^11^ manifest different degrees of depression and anxiety. However, no studies are available correlating central nervous system mental illnesses such as depression with hepatic pathology, though Traditional Chinese Medicine relates the harmful effects of rage on liver function^12^.

Metabonomics is the study of the metabolic response of living systems to genetic or environmental stimuli^13^. It provides a deeper understanding of global perturbations in metabolites and biochemical pathways in diseases, especially complex diseases^14^. Recently, metabonomics offered new insights into the pathobiology of major depressive disorders in human samples, as well as the possibility of discovering potential diagnostic^15^,^16^, therapeutic^17^, and disease biomarker candidates^18^. Chronic unpredictable mild stress (CUMS) model is a validated animal model, which produces a series of abnormal behavioral and physiological responses similar to depressive symptoms in humans^19^. Studies on metabolic disorders of CUMS-treated rat, including urine^20^,^21^,^22^,^23^,^24^,^25^,^26^,^27^, serum and plasma^28^,^29^,^30^,^31^,^32^,^33^, hippocampus^28^,^34^ and heart^7^, provide valuable insights into the mechanisms of disease and drug treatment.

Targeted liver tissue metabonomics can provide a unique perspective of the dynamic and complex metabolic changes, since liver plays a key role in a series of physiological and biochemical reactions, including synthesis, decomposition, excretion, transformation and other metabolic processes. The metabolic profile of liver tissue most closely reflects alterations in response to external stimulation and offers a novel insight into the pathology of onset and progression of depression. Metabolite fluctuations provide a real functional endpoint of cellular regulatory process and abnormal state of metabolic pathways, in contrast to the potential outcomes derived from interpretation of changes in protein and mRNA expression^35^. Thus, the combination of metabonomics-based endogenous metabolites (biomarkers) and gene expression enable the internal correlation of mRNA, protein, and metabolites with the mechanisms underlying pathogenesis and therapeutic targets of disease.

In the present study, we focus on hepatic changes in metabolites and gene expression associated with CUMS-induced depression using metabonomics and quantitative real-time polymerase chain reaction (qRT-PCR) assay. We elucidated the effect of depression on liver and provide a new perspective on its pathogenesis.

## Results

### Biochemical features

Plasma enzyme levels are a useful quantitative marker of liver injury^36^. The plasma biochemical parameters of experimental animals are summarized in Table 1. Compared with the animals in control group, the CUMS-treated rats showed significantly elevated activities of ALT and AST, suggesting that chronic unpredicted stress caused liver injury (*p* < 0.01).

### Hepatic metabolic profiles

The metabolic profiles of hepatic nonpolar and polar extracts from CUMS-treated rats were acquired using RP-UPLC-MS and HILIC-UPLC-MS in the positive and negative modes, respectively. The typical Base Peak Intensity chromatograms of the nonpolar and polar extracts from liver of the CUMS rats are shown in supporting information (Figures S1 and S2). An OPLS-DA analysis was carried out to analyze the spectral data of hepatic extracts from the control and CUMS rats. The score plots show a statistically significant difference in data between CUMS and control group in both nonpolar and polar extract (Figures S3 and S4 in supporting information), suggesting that significant biochemical changes in liver tissue occurred with CUMS treatment. The corresponding s-plots and the value of variable importance for projection (VIP > 1) were used to select altered metabolites between CUMS and control group. As a result, a total of 26 altered variables (14 detected by RP-UPLC-MS, 12 detected by HILIC-UPLC-MS) were identified in the score plot.

### Altered metabolite profile associated with CUMS-induced depression

Altered variables found in orthogonal partial least squares discriminant analysis (OPLS-DA) were identified by analysis of their molecular weight and MS^E^ spectra, and confirmed by the Databases such as Human Metabonome Database (http://www.hmdb.ca/), METLIN (http://metlin.scripps.edu/) and LIPID MAPS-Nature Lipidomics Gateway (http://www.lipidmaps.org/). The variation tendencies of the 26 identified metabolites are listed in Tables 2 and 3. Levels of 20 metabolites (**L1**-**14**, **L16**, **L19-22**, and **L25**) were increased significantly, while the others (**L15**, **L17**, **L18**, **L23, L24** and **L26**) were decreased in the CUMS-treated rats compared with the normal control.

### Potential biomarkers associated with CUMS-induced depression

A two-stage receiver operating characteristic (ROC) curve analysis was carried out to identify the potential markers associated with depression. ROC analysis is a useful tool for evaluating the accuracy of a statistical model (eg, logistic regression, linear discriminate analysis). The area under the ROC curve is a summary measure that essentially averages diagnostic accuracy.

**Figure 1:**
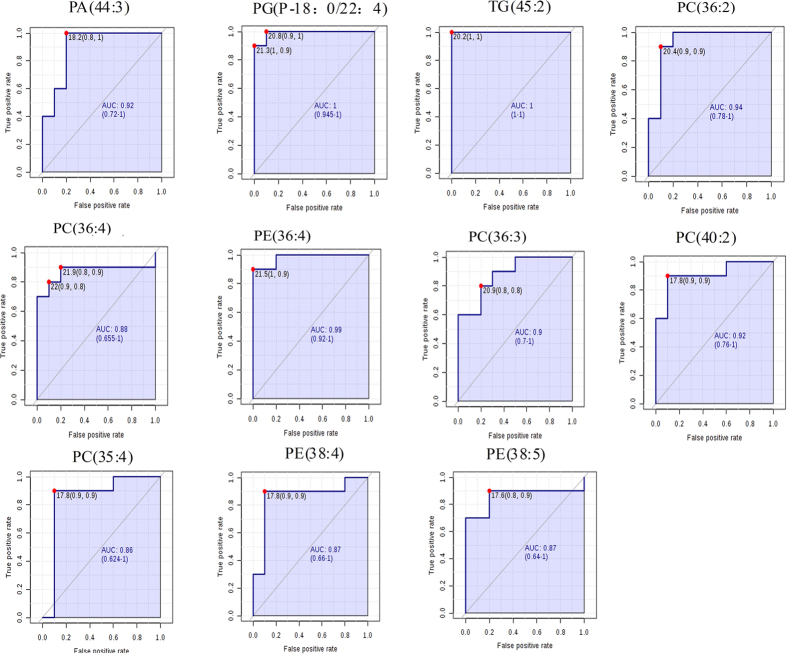
Diagnostic efficacy evaluation using ROC curves of altered metabolites of liver between CUMS-treated and Control rats detected by RP-UPLC-MS. The optimal cutoffs using the closest to top-left corner and the area under ROC curves with a 95% confidence interval were displayed (AUC > 0.85).

**Figure 2:**
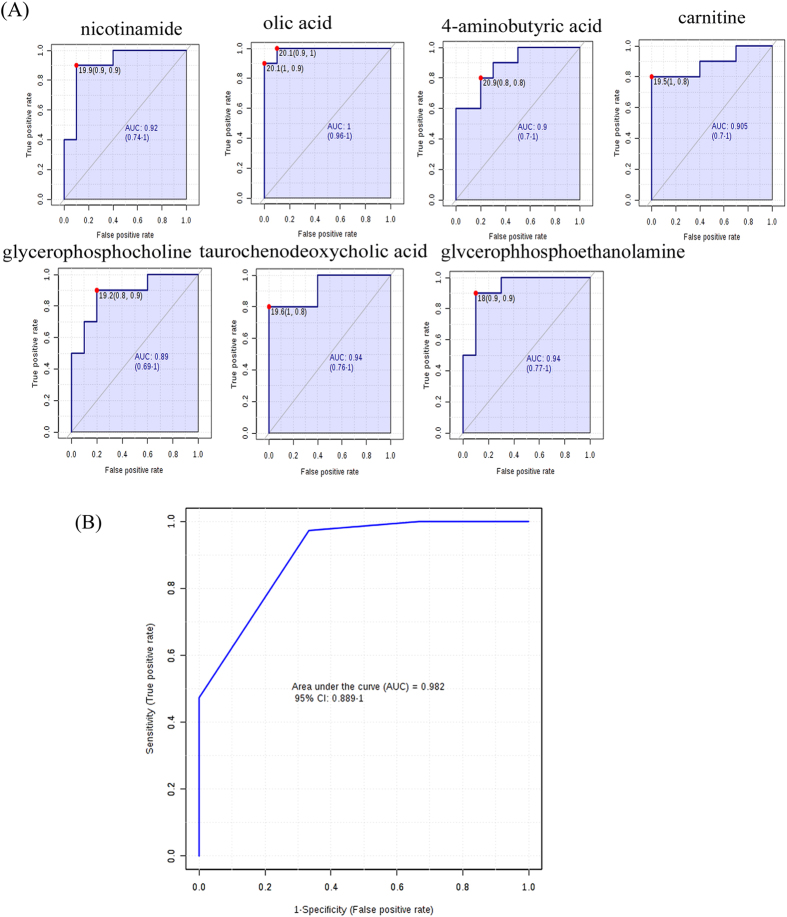
(**A**) Diagnostic efficacy evaluation using ROC curves of altered metabolites of liver between CUMS-treated and Control rats detected by HILIC-UPLC-MS. (AUC > 0.85); (**B**) ROC curve-based model evaluation (AUC = 0.982).

**Figure 3:**
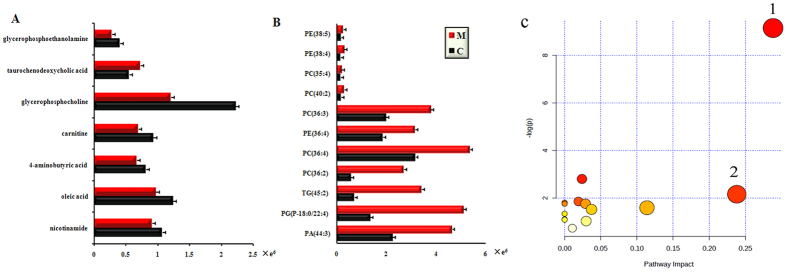
(**A**) Normalized intensity levels of potential biomarkers in liver samples of CUMS and Control group detected by RP-UPLC-MS; (**B**) normalized intensity levels of potential biomarkers detected by HILIC-UPLC-MS; (**C**) Summary of pathway analysis with MetPA. Each point represents one metabolic pathway; the size of dot and shades of color are positive correlation with the impaction of metabolic pathway. (1. Phospholipid and glycerolphospholipid systhesis; 2. Primary bile acid biosynthesis).

As a result, 18 altered metabolites with the areas under the ROC curves ranging from 0.85 to 1 (Figs 1 and 2A), were considered to show the greatest diagnostic accuracy. Subsequently, ROC curve-based model was established to assess the integrated predictive power of the combined 18 altered metabolites to distinguish depression from the normal state. The AUC value of the established model is 0.982 (Fig. 2B), which showed a good ability for discriminating CUMS-induced depression from the normal state. Thus, those 18 altered metabolites (**L1-8**, **L10-11**, **L14-15**, **L17-18**, **L23-26**) (Fig. 3A,B) can be defined as potential biomarkers associated with CUMS-induced depression.

### Perturbed metabolic pathways in response to CUMS treatment

Based on the 18 depressive potential biomarkers, a comprehensive metabolic network perturbed by CUMS treatment was mapped by KEGG (Kyoto Encyclopedia of Genes and Genomes) and MetaboAnalyst 2.0 (http://www.metaboanalyst.ca/Metabo Analyst/) (Fig. 3C). Twelve metabolic pathways were disturbed in the hepatic tissue of CUMS-treated rats. Two of the metabolic pathways with impact value >0.2 were considered as the most pertinent in CUMS-induced depression, including phospholipid metabolism and primary bile acid biosynthesis.

#### Phospholipid metabolism

Phospholipids play a dual role as basic structural components of membranes and substrates of reactions^37^,^38^. Of the two types of phospholipids (glycerophospholipids and phosphosphingolipids), glycerophospholipids are usually subdivided into phosphatidylcholine (PC), phosphatidylethanolamine (PE), phosphatidic acid (PA) and phosphoinositides. PC species is not only an essential component of biomembranes, but also protects cells and their organelles from oxidative stress, lipotoxicity, and endoplasmic reticulum (ER) stress^39^. PE species is the second most abundant membrane phospholipid in mammals, and has been identified as modulator of inflammation and apoptosis^39^. In the present work, increased levels of PC (**L4-5, L7-8, L10, and L12**), PE (**L6, L9, L11, L14**), and PA (**L1**), and reduced levels of glycerophospholipids derivatives [glycerophosphocholine (GPC) and glycerophosphoethanolamine (GPEtn), **L24** and **L26**] were detected in the liver tissue of CUMS-treated rats. The results indicated oxidative stress, inflammatory cell membrane damage, and even apoptosis in the liver during CUMS. Further, the disturbed phospholipid metabolism reduced the emulsification of bile acid, increased bile acids toxicity^40^ and further promoted liver injury.

#### Primary bile acid biosynthesis

Taurochenodesoxycholic acid (TUDCA) is a bile acid formed in the liver by conjugation of chenodeoxycholate with taurine. It is a physiological detergent that facilitates excretion, absorption, and transport of fats and sterols in the intestine and liver. TUDCA also displays hepatoprotective effect by decreasing palmitate-induced *JNK* phosphorylation, *PUMA* up-regulation and *Bax* activation, which in turn suppresses caspase-dependent hepatocyte lipoapoptosis^41^. In our experiment, enhanced level of TUDCA (**L25**) was detected in the liver of CUMS-treated rats. TUDCA accumulation might cause lipid absorption, cholesterol homeostasis and suppress hepatocyte apoptosis.

### Apoptosis-associated gene expression in CUMS liver

**Figure 4:**
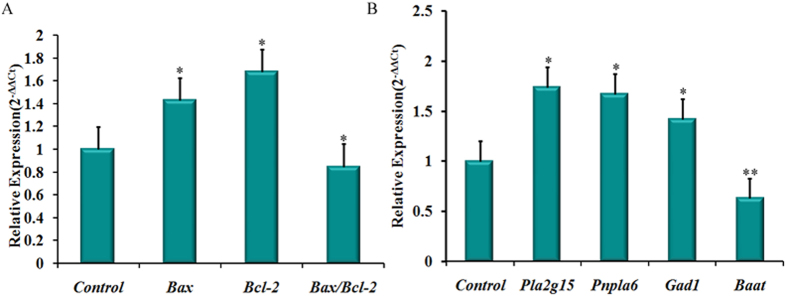
The mRNA expressions of (**A**) *Bax, Bcl-2 and Bax/Bcl-2*; (**B**) *Pla2g15, Pnpla6, Gad1* and *Baat* in liver tissues by real-time qRT-PCR assay. The expression of each target was normalized to that of the GAPDH. Statistical analysis was performed using the two-tailed Student’s t-test (n = 3) (*P < 0*.05*, **P < *0.01*).

The ALT and AST activity were significantly increased in CUMS rats, which indicated that chronic unpredicted stress caused liver injury. We examined the mRNA expression levels of apoptosis-associated genes such as *Bax* and *Bcl*-2^42^ in liver tissues by real-time qRT-PCR assay. The expression levels of *Bax* and *Bcl*-2 mRNA were 1.43 and 1.68-fold higher, and the ratio of *Bax*/*Bcl-2* mRNA was 0.85-fold lower in CUMS rats compared with normal control rats (Fig. 4A). The results indicated that unpredictable stress activated the apoptosis of hepatic cells in liver tissue.

### Key metabolic enzymes in disturbed metabolic pathways in CUMS liver tissue

Our metabonomics study showed that the hepatic phospholipids and glycerolphospholipid metabolism together with primary bile acid biosynthesis play key roles in the development of CUMS-induced depression. Thus, four key enzymes in phospholipids and primary bile acid biosynthesis, lysophospholipase I (*Pla2g15*), lysophospholipid hydrolyses (*Pnpla6*), amino acid N-acyltransferase (*Baat*) and glutamic acid dehydrogenase (*Gad1*) were selected to investigate their mRNA expression levels in the liver tissues of CUMS rats using real-time qRT-PCR assay. The results showed that the expression levels of *Pla2g15, Pnpla6*, and *Gad1* mRNA were 1.74, 1.67 and 1.42-fold higher, and *Baat* mRNA was 0.83-fold lower in CUMS rats compared with normal control (Fig. 4B). The results indicated that chronic unpredicted stimulation influenced hepatic phospholipid and glycerolphospholipid metabolism and primary bile acid biosynthesis by regulating the gene expressions of *Pla2g15, Pnpla6, Gad1* and *Baat* in liver tissue.

## Discussion

Depression is a complex psychiatric illness, which affects population of different ages. The majority of studies on depression mainly focus on changes in central nervous system, but none whatsoever on the liver tissue. Although reports show that liver disease leads to depression^7^,^8^,^9^,^10^, it is still unknown whether depression altered the liver tissue structurally and functionally. In the present study, the plasma biochemical parameters (ALT and AST) in CUMS-treated rat were significantly increased compared with normal control, which indicated that CUMS-induced depression leads to liver injury. Hepatic metabolic profiles and gene expression also indicated that CUMS-induced depression influenced liver metabolism and promoted accumulation of liver injury.

**Figure 5:**
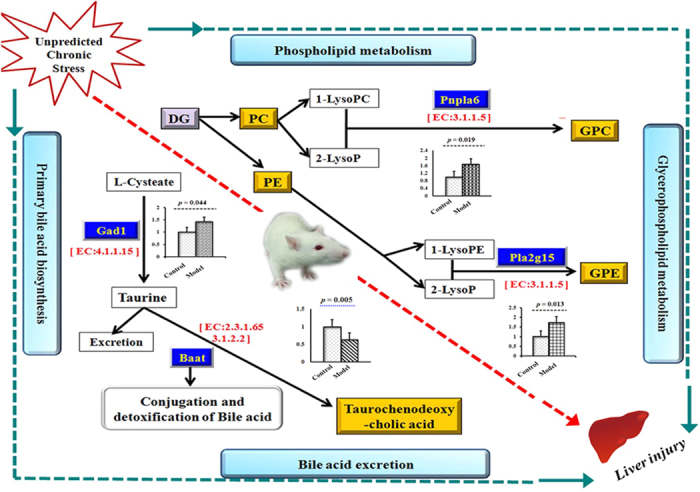
Sketch map of CUMS induced liver injury through the vital changed metabolites and variations of the corresponding hepatic genes in CUMS-treated rats. Data are expressed as mean ± S.D (n = 6 per group). Note: Yellow background represent markers found in metabonomics study. Blue background represents genes which participate in the regulation of metabolites.

Metabonomic investigation indicated that 18 potential biomarkers related to CUMS-induced depression were found in CUMS-treated rats, via twelve metabolic pathways. The phospholipid metabolism and primary bile acid biosynthesis played key roles in the development of CUMS-induced depression (Fig. 5). We also observed the mRNA expression levels of two apoptosis-associated genes (*Bax* and *Bcl*-2) to explore whether CUMS-induced depression caused liver injury. The results indicated that *Bax* and *Bcl*-2 were up-regulated, which further confirmed the impairment of hepatic cells in CUMS-induced depression.

*Bax* is a soluble protein present predominantly in the cytosol, but during the induction of apoptosis, it is translocated to mitochondrial membranes^43^. B-cell-lymphoma 2 (*Bcl-2*) protein family is comprised of antiapoptotic and proapoptotic members, which regulate the apoptosis mediated by mitochondria^44^. The ratio of *Bax/Bcl-2* in mitochondria determines the cellular response to death signals transmitted by mitochondria^45^. Zlatković *et al*.^46^ have found that the mRNA expression of *Bax* and *Bcl*-2 are decreased in rat brain under chronic isolation stress. Decreased expression of *Bcl*-2 mRNA is also reported in the hippocampus and prefrontal cortex of prenatally stressed offspring rats^47^. In contrast to the results in the brain, we observed an increased expression of *Bax* and *Bcl*-2 mRNA in the liver tissue of CUMS-treated rats in the present study. Similar increased expression of *Bax* in liver was also found in acute CCl_4-_induced liver damage^48^,^49^ which suggested that chronic unpredictable stress activated the apoptosis of hepatic cells in liver tissue. The increased expression of *Bcl*-2 mRNA and the down-regulated expression of *Bax*/*Bcl*-2 ratio indicated that antiapoptotic and proapoptotic effects occurred simultaneously, along with a higher mRNA expression of restraining apoptosis gene (*Bcl*-2). The present results suggested that CUMS-induced depression activated the apoptosis of hepatic cells and the liver tissue underwent a compensatory protective state.

Further, the gene expressions of key enzymes (*Pla2g15, Pnpla6, Gad1* and *Baat*) involved in phospholipids and primary bile acid metabolism were also identified by real-time qRT-PCR assay. The results confirmed the biochemical changes observed in metabonomics study, indicating the variations of the downstream endogenous metabolites were correlated with the upstream gene expression.

*Pla2g15* acts on biological membranes to regulate the multifunctional lysophospholipids. The protein encoded by this gene hydrolyzes lysophosphatidylcholine (LysoPC) to glycerophosphocholine (GPC) and a free fatty acid. It plays a role in degradation of phospholipids^38^. Patatin-like phospholipase domain-containing protein 6 (*Pnpla6*) is a neuropathy target esterase enzyme that deacetylates intracellular phosphatidylcholine (PE) to produce glycerophosphocholine (GPC) (Fig. 5). Phospholipids are a class of lipids that are a major component of all cell membranes as they can form lipid bilayers. Phosphatidylcholine (PC) and phosphatidylethanolamine (PE) are two major phospholipids that are asymmetrically distributed in the plasma membrane: the majority of PC is localized to the outer leaflet, whereas PE is enriched in the inner leaflet^39^. The observed increase in PC or PE may be linked to structural membrane changes, since it is a cone-shaped phospholipid with a small head group introducing tension into membrane bilayers and also potentially increasing permeability of the plasma membrane^50^,^51^,^52^. Li *et al*.^50^ reported that liver damage might alter phospholipid levels in the plasma membrane and reduce plasma membrane integrity in choline-deficient (CD) mouse model. In the present work, the increased levels of PC and PE (**L4-12**, **L14)** were observed, which suggested that CUMS might cause liver damage by altering the structural integrity and permeability of plasma membrane in liver tissue. We observed the increased expression levels of *Pla2g15* and *Pnpla6* mRNA (Fig. 5) of liver after CUMS treatment using qRT-PCR. The present study suggested that the body might lower the high expression of *Pla2g15* and *Pnpla6* abnormally in PC or PE and correct the abnormality of phospholipid metabolism. However, reduced levels of GPC and GPEtn (**L24** and **L26**), which are the glycerophospholipid derivatives were also observed in our work. The inconsistent mRNA expression and metabolites is perhaps due to PC and PE transformation into other metabolites (1, 2-diacyl-sn-glycerol and 1-acyl-sn-glycero-3-phosphocholine and 2-acyl-sn-glycero-3-phosphocholine) except that GPC and GPE are mediated via other pathways or regulated by other genes (*LCAT, Pla2g16* and *plcC, etc*). Therefore, the present work confirmed that a drastic shift occurred in the balance of phospholipid synthesis and chronic unpredictable mild stress caused liver damage via altered structural integrity and permeability of plasma membrane in liver tissue.

*Gad1* and *Baat* are vital genes in bile acid synthesis and excretion in rat liver tissues. *Gad1* encodes one of the several forms of glutamic acid decarboxylase. It catalyzes the production of taurine from L-cysteine in liver tissue^43^ (Fig. 5). *Baat* is the sole enzyme responsible for conjugation of primary and secondary bile acids to taurine and glycine in primary bile acid synthesis. The conjugates of bile acid, TUDCA (**L25**) and GLCA, act as detergents in the gastrointestinal tract, to enhance lipid and fat-soluble vitamin absorption. In the present work, the increased level of *Gad1* and decreased level of *Baat* mRNA suggested dysfunction in bile acid synthesis and excretion in liver tissue of CUMS-treated rats. We only found increased TUDCA (**L25**), which plays a hepatoprotective role through suppressed caspase-dependent hepatocyte lipoapoptosis^41^. The increased level of TUDCA suggested a self-protection mechanism through secretion of large amounts of TUDCA. The inconsistent gene expression and altered metabolite content might be related to liver compensatory action in response to chronic stress to maintain normal metabolism. In addition, the down-regulation of *Baat* might cause the dysfunction of bile acid synthesis and excretion, which further promote bile acid toxicity in the liver and reduce lipid metabolite absorption during the process of CUMS.

In summary, we found that CUMS-induced depression caused liver injury, with a drastic shift in the phospholipid metabolism and bile acid synthesis (Fig. 5), and activated hepatocyte apoptosis. Drastic changes in the metabolite levels suggested that the ultimate response of biological systems to genetic or environmental changes can be observed in the end products of cellular regulatory processes. The current study revealed that depression caused hepatic changes in metabolite levels and gene expression, even though further functional confirmation of potential biomarkers and underlying mechanism is needed.

## Conclusions

In the current study, hepatic metabolic profile and gene expression were performed by HILIC-UPLC and RP-UPLC coupled with mass spectrometry and real-time qRT-PCR to investigate the liver response to CUMS-induced depression. A panel of 18 altered metabolites was identified as potential biomarkers related to depression via a two-stage ROC analysis of metabonomics data. Abnormal phospholipid and bile acid biosynthesis were the major metabolic events in the rat models during chronic unpredictable mild stress. The increased ALT and AST and increased expression levels of apoptosis-associated genes such as *Bax* and *Bcl*-2, and the down-regulated expression of *Bax*/*Bcl*-2 ratio in liver tissues indicated that CUMS activated the apoptosis of hepatic cells and caused liver injury. Furthermore, the expression levels of the key enzymes in phospholipids and primary bile acid metabolism, including *Pla2g15, Pnpla6, Gad1* and *Baat* were triggered in liver tissue by CUMS treatment. The results suggest that the accumulated variations in hepatic metabolites ultimately resulted in liver injury following depression. Our findings provide a new perspective on the pathogenesis of depression.

## Methods

### Chemicals and reagents

HPLC-grade methanol and acetonitrile were purchased from Merck (Darmstadt, Germany). The water used for UPLC was purified by a Milli-Q system (Millipore, France). Formic acid (HPLC grade) was obtained from Tedia (Fairfield, USA). Leucine-enkephalin was obtained from Sigma Aldrich (St. Louis, MO, USA). The assay kits for alanine aminotransferase (ALT) and aspartate aminotransferase (AST) were purchased from Nanjing Jiancheng Bioengineering Institute (Nanjing, China). Ultrapure water (18.2 MΩ) was prepared with a Milli-Q water purification system (Millipore, France). All the other chemicals used were of analytical grade. TRIzol Reagent was obtained from Invitrogen. PrimeScript™ RT reagent Kit and SYBR premix Ex Taq™ II kit (TliRNaseH Plus) were purchased from TaKaRa.

### Animal treatment and sample collection

Twenty healthy, adult, male Wistar rats, weighting 200 ± 20 g each, were purchased from the Institute of Laboratory Animal Science, CAMS & PUMC (Beijing, China). The rats were housed individually in cages for one week to adapt to the environment under controlled conditions of 12 h light-12 h dark cycles (lights on from 6:00 a.m.–6:00 p.m.), 10% relative humidity and temperature (20 ± 3 °C) with commercial diet and water available *ad libium*. The rats were randomly divided into two groups: (1) Control group, (2) CUMS group. Untreated rats served as the control group, and the CUMS-treated rats were subjected to a series of variable stimuli as previously described^28^.

All rats were anaesthetized using sodium pentobarbital on the 28^th^ day. Blood samples were collected, and centrifuged at 3000 *rpm* for 15 min at 4 °C. The liver tissues were quickly removed and frozen in liquid nitrogen and kept at −80 °C until analysis. One part of liver tissue was used for the metabonomic study, and the other part was used for the qRT-PCR analysis.

The study was approved by the Ethics Committee of the Institute of Medicinal Plant Development, CAMS&PUMC. All experimental procedures were performed in accordance with relevant guidelines approved by the Ethics Committee of the Institute of Medicinal Plant Development, CAMS&PUMC.

### Clinical chemistry analysis

Biochemical indexes of plasma including ALT and AST were determined by spectrophotometry (Mapada, UV-3100, China) using enzymatic standard kits (Nanjing JianchengInstitute of Biotechnology) according to the manufacturer’s instructions.

### Sample preparation

A two-step extraction procedure was carried out according to the Pan’s method with some modification^53^. For each 250 mg tissue sample, 1500 *μ*L of chilled methanol-water (5:1, v/v) was added to the tissue sections in a tube. The samples were homogenized keeping in an ice-bath with a homogenizer (IKA, Staufen, Germany) for 30 s. Then the homogenate was centrifuged at 13000 *rpm* for 15 min at 4 °C. The supernatant was transferred to a fresh tube and used as polar solvent extracts. And then the deposit was homogenized with chloroform-methanol (4:1, v/v), 1500 *μ*L under the same operation process. The supernatant was transferred to another fresh tube and used as nonpolar solvent extracts. Two kind samples were dried under N_2_ and stored at −80 °C until LC-MS analysis.

The dried residue of nonpolar solvent extracts was dissolved in 500 *μ*L of acetonitrile-water (2:8, v/v), and 5 *μ*L were injected into the UPLC system for analysis by BEH C18 column after centrifugation for 20 min at 13000 *rpm*. And the dried residue of polar solvent extracts was dissolved in 500 *μ*L of acetonitrile-water (1:1, v/v) and 5 *μ*L were injected into the UPLC system for analysis by HILIC column (100 mm × 2.1 mm, 1.7 *μ*m) after centrifugation for 20 min at 13000 *rpm*.

### UPLC-Q-TOF/MS analysis

#### Method development

Chromatographic separation was performed on Waters ACQUITY UPLC System (Waters Corp. Milford, USA), equipped with a binary solvent delivery system, and an autosampler. The nonpolar extracts of liver sample was performed on an Acquity UPLC BEH C18 column (100 mm × 2.1 mm, 1.7 *μ*m) and the polar extracts of liver sample was performed on an Acquity UPLC BEH HILIC column (100 mm × 2.1 mm, 1.7 *μ*m). The columns were maintained at 40 °C and eluted at a flow rate of 0.40 mL/min, using a mobile phase of (A) 0.1% (by volume) formic acid in water and (B) acetonitrile. The gradient program for nonpolar extracts of liver was optimized as follows: 10% B from 0 to 1 min, 50–95% B from 1 to 6 min, 95–98% B from 6 min to 8 min, and 100% B from 8 to 12 min. The gradient program for polar extracts of liver was optimized as follows: 99% B from 0 to 1 min, 99–70% B from 1 to 10 min, and 99% B from 10 to 15 min.

Mass spectrometry data were collected using a Q-TOF analyzer in a SYNAPT HDMS system (Waters Corporation, Milford, MA, USA) in both positive and negative ion modes. The source temperature was set at 120 °C with a cone gas flow of 50 L/H, a desolvation gas temperature of 300 °C and a desolvation gas flow of 800 L/H. For the positive and negative ion modes, the capillary voltage was set to 3.0 kV and 2.5 kV, respectively, and the cone voltage was set to 35 V. The mass spectrometric data were collected in centroid mode from *m/z* 50 to 1200 with a scan time of 0.3 s and an interscan delay of 0.02 s over a 15 min analysis time. Leucine-enkephalin was used as the lock mass (*m/z* 556.2771 in positive mode and *m/z* 554.2615 in negative mode) at a concentration of 0.5 *μ*g/mL with a flow rate of 80 *μ*L/min The lock spray frequency was set at 20 s and the lock mass data were averaged over 10 scans for correction.

##### Method Validation

To ensure the stability of sequence analysis, a quality control (QC) sample was prepared by pooling the same volume (100 *μ*L) from each prepared liver sample and then preparing the pooled QC sample in the same way as the samples. To provide assurance that the system was suitable for use, five pooled QC samples were run prior to analysis. And these pooled QC samples were interspersed between every five biological samples during the analytical run. Ten ions were extracted from the Base Peak Intensity chromatography and selected for method validation. The relative standard deviations (R.S.D) of retention time and *m/z* of the selected ions from RP-UPLC-MS and HILIC-UPLC-MS were list in Tables S1 and S2, respectively (shown in Supplementary). Method repeatability was evaluated using six replicates by analyzing the QC sample. The RSD of retention times and *m/z* of the selected ions in positive mode and negative mode were listed in Tables S3 and S4 (shown in supplementary). The reproducibility of extraction process was investigated using healthy rat liver tissue. Reproducibility was analyzed by repeating the whole method for the same live tissue within a day using LC-MS (n = 6). For most of the compounds investigated, the peak area RSD values of reproducibility were below 10% (Supplementary Table S5).

### Data Processing

The raw data were analyzed using the MarkerLynx Applications Manager version 4.1 (Waters, Manchester, U.K.), which allowed for deconvolution, alignment and data reduction to provide a list of mass and retention time pairs with corresponding intensities for all of the detected peaks from each data file in the data set.

Multivariate data analysis (MVA) was performed using Simca-p software (v12.0, Umetric, Umeå, Sweden). Imported data set were normalization, mean-centered and pareto-scaled prior to multivariate analysis. Principal components analysis (PCA) and orthogonal partial least squares discriminate analysis (OPLS-DA) were employed to process the acquired MS data. PCA was performed to discern the natural separation between different stages of samples by visual inspection of score plots. In the OPLS-DA model, samples from different groups were classified, and the results were visualized in the form of score plots to show the group clusters and S-plots to show the variables that contributed to the classification.

The receiver operating characteristic (ROC) curve was performed to evaluate the accuracy of identified metabolites in distinguishing depression from control by using a web-based tool called ROCCET (ROC Curve Explorer, http://www.roccet.ca)^54^.

### RNA Isolation and Quantitative Real-time Polymerase Chain Reaction (qRT-PCR)

Liver tissues were collected and stored in liquid nitrogen until use. Each sample of total RNA was extracted from 100 mg tissues using the TRIzol Reagent. RNA integrity was analyzed on a 1.2% agarose gel. RNA quantity was determined using a NanoDrop 2000C Spectrophotometer (Thermo Scientific). 1 *μ*g of RNA was reverse-transcribed with a PrimeScript™ RT reagent Kit (TaKaRa) for cDNA synthesis and genomic DNA removal. The qPCRs were performed according to the instructions of the SYBR premix Ex Taq™ II kit (TliRNaseH Plus) (TaKaRa) and carried out in triplicate using the CFX96™ real-time PCR detection system (Bio-Rad). Reaction conditions were: 30 s at 95 °C, followed by 40 cycles of 95 °C for 5 s, and 60 °C, 30 s. Gene-specific primers were designed using online primer designing tools primer-blast (http://www.ncbi.nlm.nih.gov/tools/primer-blast/) and synthesis in Invitrogen. The primer sequences are listed in supporting information (Table S6). The lengths of amplifications are between 100 bp and 250 bp. GAPDH was chosen as an endogenous control in this study. Standard deviations were calculated from three PCR replicates. The specificity of amplification was assessed by dissociation curve analysis, and the relative abundance of genes was determined using the comparative Ct method as suggested by the CFX-manager software (Bio-Rad).

### Statistical analysis

All values were expressed as mean ± S.D. The significance of differences between the means of the CUMS and control group has been compared by two-tailed *Student’s t-test* using the Statistical Package for Social Science program (SPSS 16.0, SPSS, Chicago, IL, USA). The significance threshold was set at *p* < 0.05 for this test.

## Additional Information

**How to cite this article**: Jia, H.M. *et al*. Chronic unpredictive mild stress leads to altered hepatic metabolic profile and gene expression. *Sci. Rep.* **6**, 23441; doi: 10.1038/srep23441 (2016).

## Supplementary Material

Supplementary Information

## Figures and Tables

**Table 1:** Plasma biochemical parameters in experimental rats (values are given as mean ± S.D, n = 6).

	ALT(IU/L)	AST(IU/L)
Control group	16.90 ± 2.77	128.42 ± 21.48
CUMS treated group	203.36 ± 32.72^**^	432.62 ± 67.69^**^

**p < 0.01 compared with control group.

**Table 2:** The altered metabolites detected by RP-UPLC-MS of non-polar extracts of liver from CUMS-treated rat and their variation tendency.

NO.	Metabolites	Rt _Mass	VIP	Trend	Metabolic pathway
**L1**	PA(44:3)^+^	6.53_810.6000	5.4	↑^**^	Phospholipid metabolism
**L2**	PG(P-18:0/22:4)^+^	6.95_810.5998	4.7	↑^*^	Phospholipid metabolism
**L3**	TG(45:2)^+^	7.97_760.5845	10.2	↑^**^	Lipid metabolism
**L4**	PC(36:2)^+^	8.17_786.6004	6.5	↑^**^	Phospholipid metabolism
**L5**	PC(36:4)^+^	11.03_782.5687	6.3	↑^**^	Phospholipid metabolism
**L6**	PE(36:4)^+^	11.24_740.5218	5.9	↑^**^	Phospholipid metabolism
**L7**	PC(36:3)^+^	11.65_784.5839	11.2	↑^**^	Phospholipid metabolism
**L8**	PC(40:2)^−^	7.13_878.5911	6.5	↑^**^	Phospholipid metabolism
**L9**	PE(44:2)^−^	8.94_854.5913	22.4	↑^**^	Glycerophospholipid metabolism
**L10**	PC(35:4)^−^	9.41_766.5380	14.5	↑^*^	Glycerophospholipid metabolism
**L11**	PE(38:4)^−^	9.67_766.5385	8.5	↑^**^	Glycerophospholipid metabolism
**L12**	PC(20:3/P-16:0)^−^	9.72_804.5754	4.3	↑^**^	Glycerophospholipid metabolism
**L13**	PG(34:1)^−^	11.38_747.5657	5.8	↑^**^	Glycerolipid metabolism
**L14**	PE(38:5)^−^	12.07_764.5224	7.1	↑^*^	Glycerophospholipid metabolism

“+” represent the metabolites detected in positive mode;

“−” metabolites detected in negative mode.

*p < *0.05*, **p < *0.01* com*p*ared with control group.

**Table 3:** The altered metabolites detected by HILIC-UPLC-MS of polar extracts of liver from CUMS-treated rat and their variation tendency.

NO.	Metabolites	Rt_Mass	VIP	Trend	Metabolic Pathway
L15	nicotinamide^+^	1.15_123.0560	6.2	↓^**^	Nicotinate and nicotinamide metabolism
L16	hypoxanthine^+^	1.62_137.0463	9.5	↑^**^	Purine metabolism
L17	oleic acid^+^	4.02_283.1757	7.3	↓^**^	Biosynthesis of unsaturated fatty acids
L18	4-aminobutyric acid^+^	4.85_104.1076	6.9	↓^**^	Alanine, aspartate and glutamate metabolism
L19	isovalerylcarnitine^+^	5.45_246.1704	5.4	↑^**^	Lysine degradation
L20	isobutyryl-1-carnitine^+^	5.6_232.1539	5.7	↑^**^	Lysine degradation
L21	O-Acetyl carnitine^+^	6.00_204.1236	10.9	↑^**^	Lysine degradation
L22	creatine^+^	6.14_132.0775	7.0	↑^**^	Glycine, serine and threonine metabolism
L23	carnitine^+^	6.23_162.1130	26.2	↓^**^	Lysine degradation
L24	glycerophosphocholine^+^	8.25_258.1110	13.5	↓^**^	Glycerophospholipid metabolism
L25	taurochenodeoxycholic acid^−^	0.88_498.2882	9.4	↑^**^	Primary bile acid biosynthesis
L26	glycerophosphoethanolamine−	7.07_214.0481	3.9	↓^**^	Glycerophospholipid metabolism

“+” represent the metabolites detected in positive mode;

“−” metabolites detected in negative mode.

*p < *0.05,* **p < *0.01* compared with control group.
