# Supplementary material for: Chronic unpredictive mild stress leads to altered hepatic metabolic profile and gene expression
Source: Sci Rep. 2016 Mar 23;6:23441. doi: 10.1038/srep23441 (PMC4804211; doi:10.1038/srep23441)
Supplement: Supplementary Information [file srep23441-s1.pdf]

Chronic unpredictive mild stress leads to altered hepatic metabolic profile and gene expression

Hong-mei Jia[#], Qi Li, Chao Zhou, Meng Yu, Yong Yang, Hong-wu Zhang, Gang Ding, Hai Shang, Zhong-mei Zou[*]

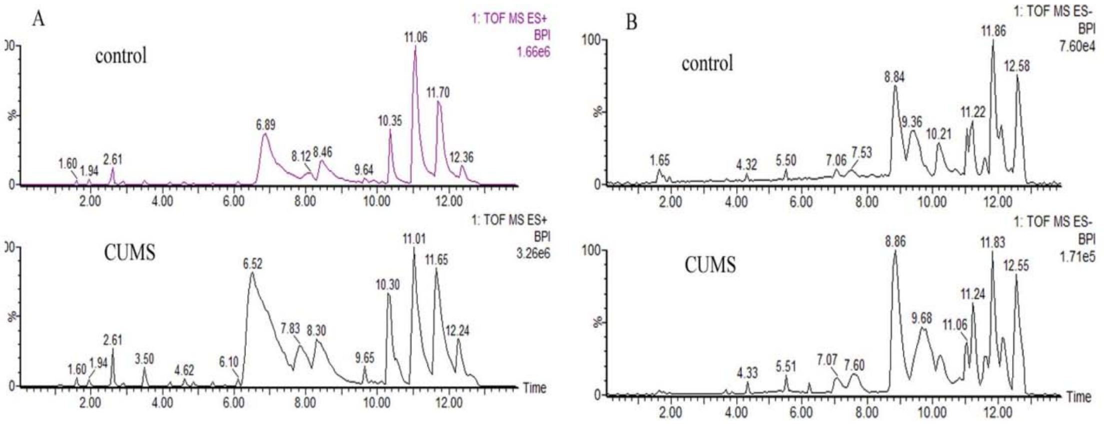

Figure S1. The typical Base Peak Intensity chromatograms of the nonpolar solvent extracts of liver tissues from control and CUMS-treated rats detected by RP-UPLC-MS. A: Positive mode; B: Negative mode.

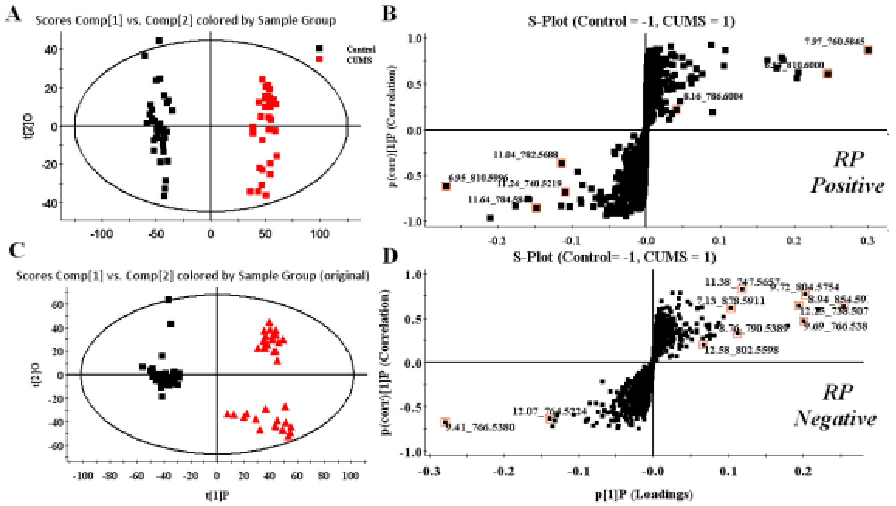

Figure S2. OPLS-DA analysis of data obtained from the nonpolar solvent extracts of liver tissue detected by RP-UPLC-MS from control and CUMS-treated rats. (A, B) Scores plots and S-plots of OPLS-DA model in positive mode (R^2X=0.398, R^2Y=1, $Q^2(cum)$=0.976); (C, D) Scores plots and S-plots of OPLS-DA model in negative mode (R^2X=0.35, R^2Y=1, $Q^2(cum)$=0.896); Symbols, control rat (black) and CUMS-treated rat (red).

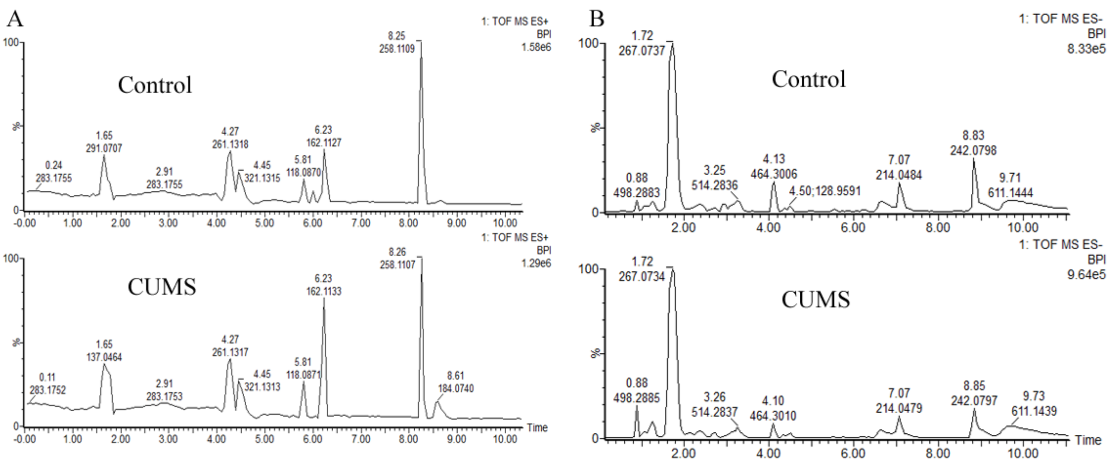

Figure S3. The typical Base Peak Intensity chromatograms of the polar extracts of liver tissues from control and CUMS-treated rats detected by HILIC-UPLC-MS

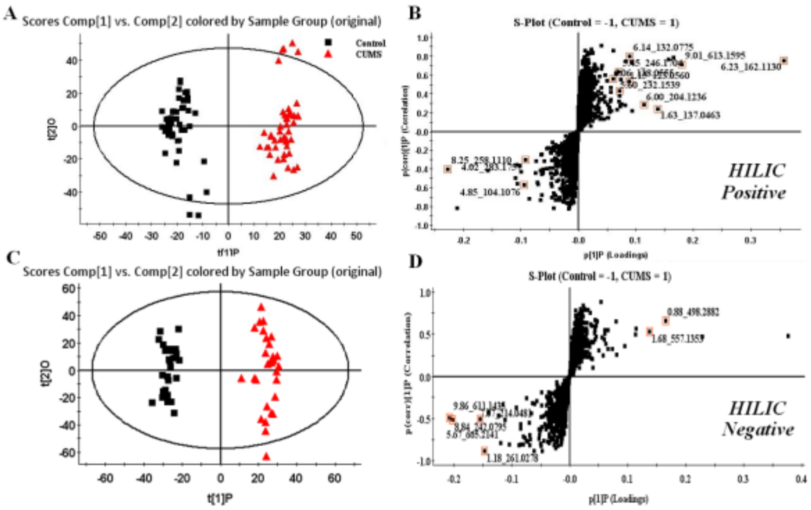

Figure S4. OPLS-DA analysis of data obtained from the polar solvent extracts of liver tissue detected by HILIC-UPLC-MS from control and CUMS-treated rats. (A, B) Scores plots and S-plots of in positive mode (R^2X=0.366; R^2Y=1, $Q^2(cum)$=0.966); (C, D) Scores plots and S-plots detected by HILIC-MS in negative mode (R^2X=0.552, R^2Y=1, $Q^2(cum)$=0.964). Symbols, control rat (black) and CUMS-treated rat (red).

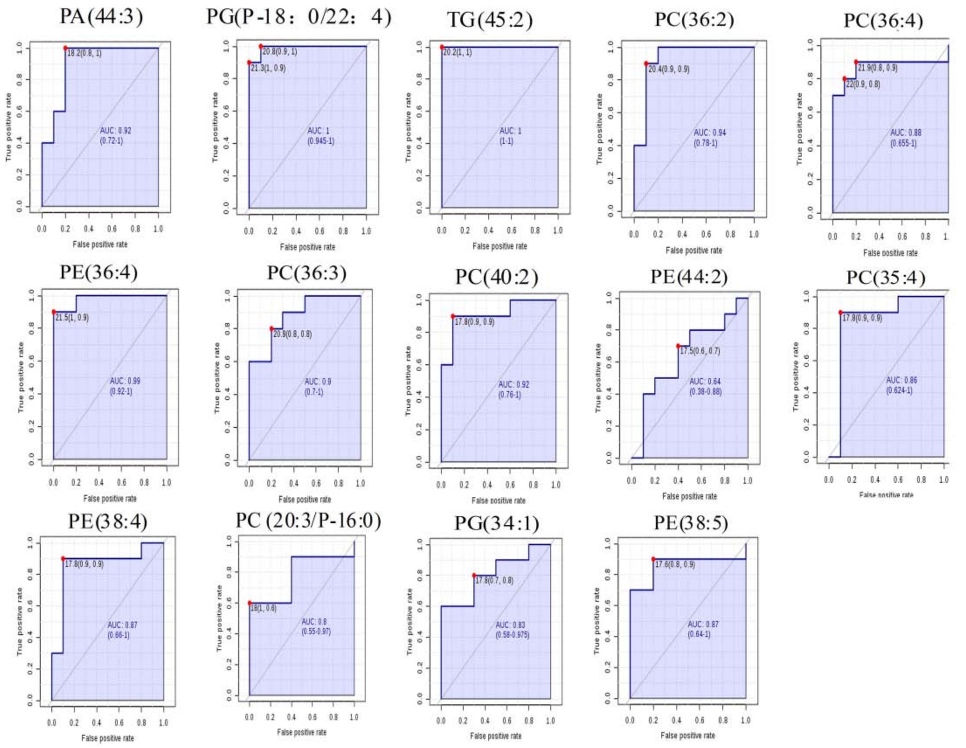

Figure S5 Diagnostic efficacy evaluation using ROC curves of metabolites detected by RP-UPLC-MS .The optimal cutoffs using the closest to top-left corner and the area under ROC curves with a 95% confidence interval were displayed.

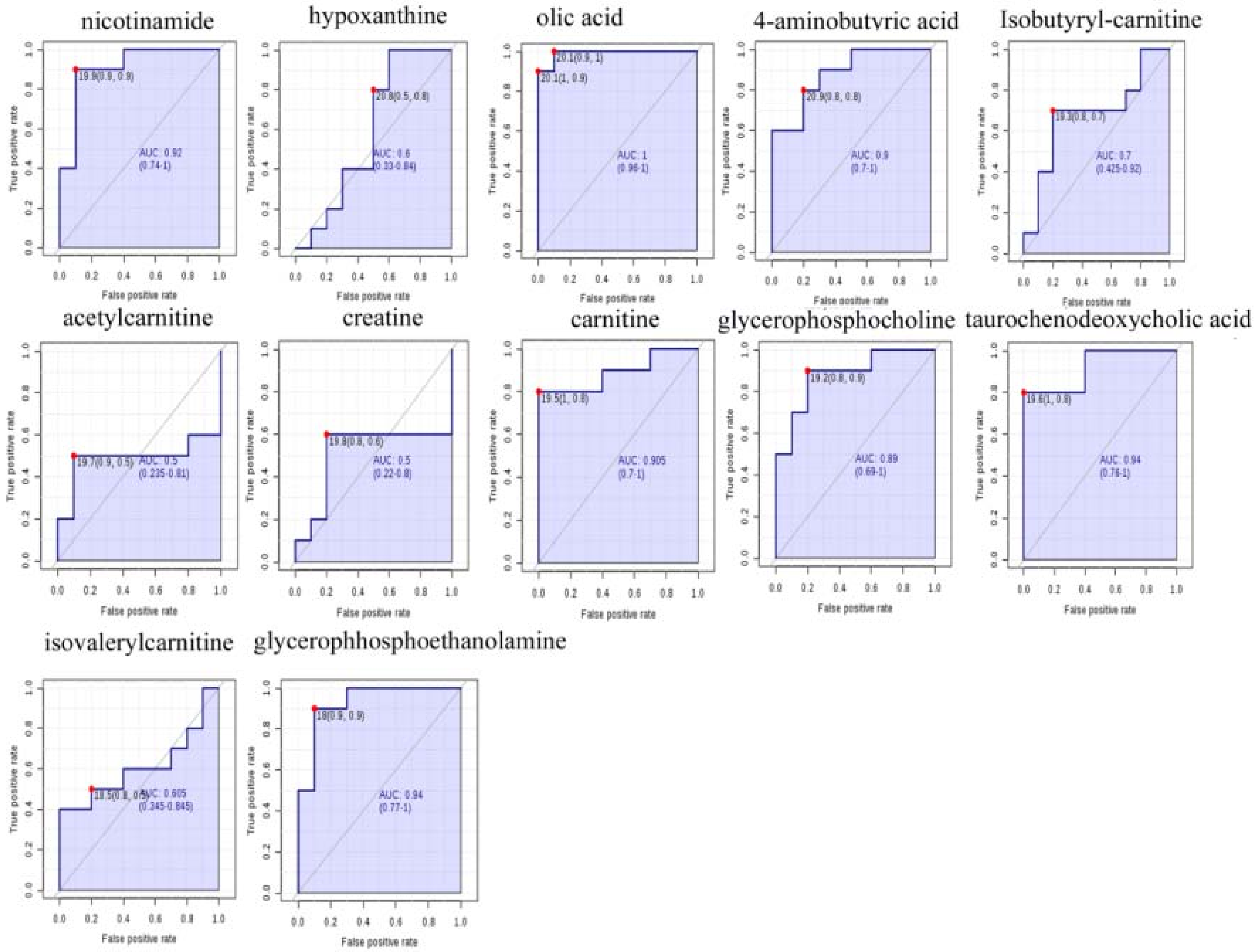

Figure S6 Diagnostic efficacy evaluation using ROC curves of metabolites detected by HILIC-UPLC-MS. The optimal cutoffs using the closest to top-left corner and the area under ROC curves with a 95% confidence interval were displayed

The stability and repeatability of UPLC-Q-TOF/MS method validation under the positive and negative ion modes using QC sample.

Table S1. Stability of nonpolar extracts of liver tissue detected by RP-MS method

NO.	Positive mode				NO.	Negative mode			
	RT (min)	RSD (%)	*m/z*	RSD (%)		RT (min)	RSD (%)	*m/z*	RSD (%)
1	1.60	0.0045	218.2204	0.0039	11	1.65	0.0015	181.0154	0.0003
2	2.61	0	274.2742	0.0061	12	1.94	0.0026	218.0121	0.0008
3	3.55	0.0071	302.3052	0.0065	13	3.67	0.0103	588.3319	0.0014
4	4.21	0.0045	496.3393	0.0085	14	4.32	0.0129	540.3304	0.0007

5	5.40	0.0045	524.3704	0.0094	15	5.46	0.0062	568.3619	0.0006
6	8.11	0.0084	786.6029	0.0137	16	6.18	0.0055	681.2959	0.0008
7	9.59	0.0179	806.5721	0.0146	17	6.94	0.0079	790.5385	0.0002
8	10.29	0.0207	806.5728	0.0141	18	8.94	0.0164	766.5394	0.0008
9	10.93	0.0122	782.572	0.0139	19	11.54	0.0271	826.5598	0.0004
10	11.59	0.0207	758.5706	0.0134	20	12.21	0.0222	802.5603	0.0005

Table S2. Repeatability of nonpolar extracts of liver tissue detected by RP-UPLC-MS method

NO.	Positive mode				NO.	Negative mode			
	RT (min)	RSD (%)	*m/z*	RSD (%)		RT (min)	RSD (%)	*m/z*	RSD (%)
1	1.60	0.0065	218.2204	0.0025	11	1.65	0.0033	181.0154	0.0011
2	2.61	0.0034	274.2742	0.0132	12	1.94	0.0015	218.0121	0.0004
3	3.55	0.0084	302.3052	0.0083	13	3.67	0.0087	588.3319	0.0062
4	4.21	0.0066	496.3393	0.0067	14	4.32	0.0043	540.3304	0.0012
5	5.40	0.0043	524.3704	0.0121	15	5.46	0.0089	568.3619	0.0004
6	8.11	0.0165	786.6029	0.0105	16	6.18	0.0121	681.2959	0.0011
7	9.59	0.0087	806.5721	0.0232	17	6.94	0.0076	790.5385	0.0007
8	10.29	0.0133	806.5728	0.0044	18	8.94	0.0105	766.5394	0.0023
9	10.93	0.0095	782.572	0.0097	19	11.54	0.0035	826.5598	0.0009
10	11.59	0.0011	758.5706	0.0211	20	12.21	0.0143	802.5603	0.0010

Table S3. Stability of polar extracts of liver tissue detected by HILIC-UPLC-MS method

NO.	Positive mode				NO.	Negative mode			
	RT (min)	RSD (%)	*m/z*	RSD (%)		RT (min)	RSD (%)	*m/z*	RSD (%)
1	1.65	0.0179	291.0701	0.0002	11	0.88	0.0042	498.2888	0.0003
2	3.26	0.0203	274.2744	0.0002	12	2.60	0.0133	282.0844	0.0002
3	3.43	0.0199	318.2998	0.0002	13	3.48	0.0143	514.2836	0.0006
4	4.15	0.0163	261.1307	0.0002	14	4.20	0.0163	464.3013	0.0002
5	4.57	0.0063	321.1313	0.0001	15	4.69	0.0178	128.959	0.0002
6	5.28	0.0065	728.5701	0.0017	16	5.30	0.0229	245.0426	0.0003
7	6.21	0.0149	204.1232	0.0001	17	6.04	0.0085	323.0283	0.0003

8	6.50	0.0149	162.1125	0.0001	18	7.50	0.0279	214.0482	0.0001
9	6.81	0.0152	261.1429	0.0004	19	9.39	0.0198	242.0796	0.0141
10	9.15	0.0196	184.0736	0.0002	20	10.14	0.0101	611.1443	0.0003

Table S4. Repeatability polar extracts of liver tissue detected by HILIC-UPLC-MS method

NO.	Positive mode				NO.	Negative mode			
	RT (min)	RSD (%)	*m/z*	RSD (%)		RT (min)	RSD (%)	*m/z*	RSD (%)
1	1.65	0.0065	291.0701	0.0004	11	0.88	0.0033	498.2888	0.0012
2	3.26	0.0114	274.2744	0.0003	12	2.60	0.0078	282.0844	0.0007
3	3.43	0.0098	318.2998	0.0011	13	3.48	0.0121	514.2836	0.0011
4	4.15	0.0211	261.1307	0.0009	14	4.20	0.0045	464.3013	0.0009
5	4.57	0.0066	321.1313	0.0002	15	4.69	0.0133	128.959	0.0001
6	5.28	0.0171	728.5701	0.0006	16	5.30	0.0155	245.0426	0.0005
7	6.21	0.0096	204.1232	0.0004	17	6.04	0.0172	323.0283	0.0009
8	6.50	0.0218	162.1125	0.0010	18	7.50	0.0099	214.0482	0.0021
9	6.81	0.0173	261.1429	0.0004	19	9.39	0.0044	242.0796	0.0044
10	9.15	0.0044	184.0736	0.0003	20	10.14	0.0039	611.1443	0.0011

Table S5. Reproducibility of the extraction process based on UPLC-Q/TOF MS

RP-UPLC-MS								HILIC-UPLC-MS							
NO.	Positive mode			NO.	Negative mode			NO.	Positive mode			NO.	Negative mode		
	RT (min)	*m/z*	RSD (%)		RT (min)	*m/z*	RSD (%)		RT (min)	*m/z*	RSD (%)		RT (min)	*m/z*	RSD (%)
1	1.60	291.0701	10.2539	11	1.65	181.0154	12.0000	1	1.65	291.0701	11.8864	11	0.88	498.2888	12.4967
2	2.61	274.2744	9.2495	12	1.94	218.0121	11.9038	2	3.26	274.2744	13.1022	12	2.60	282.0844	9.54289
3	3.55	318.2998	12.8591	13	3.67	588.3319	13.9392	3	3.43	318.2998	9.5019	13	3.48	514.2836	10.9483
4	4.21	261.1307	12.6371	14	4.32	540.3304	10.9179	4	4.15	261.1307	8.4825	14	4.20	464.3013	9.04249
5	5.40	321.1313	5.7071	15	5.46	568.3619	12.8258	5	4.57	321.1313	11.0862	15	4.69	128.959	11.7587
6	8.11	728.5701	9.3350	16	6.18	681.2959	11.4543	6	5.28	728.5701	10.5627	16	5.30	245.0426	8.92188
7	9.59	204.1232	10.5661	17	6.94	790.5385	11.3666	7	6.21	204.1232	11.0970	17	6.04	323.0283	10.7842
8	10.29	162.1125	14.3800	18	8.94	766.5394	9.15423	8	6.50	162.1125	13.1891	18	7.50	214.0482	8.18942
9	10.93	261.1429	9.2341	19	11.54	826.5598	10.2078	9	6.81	261.1429	12.6416	19	9.39	242.0796	10.8751

Gene	Sequence 5’-3’	Length	Product	Tm	GC%
Pla2g15	S: TGAATGGGTTTTGGTTTGCG	20	89	61.7	45
	A: AAGCCCCTAGCTGGGACTCTAA	22		61.5	54.5
Pnpla6-1	S: CTGGTCACCGCCGTACTCAT	20	140	60.6	60.0
	A: GACACCTTCCGCATAATCTTCC	22		60.4	50.0
Gad1	S: GGCACGACTGTTTATGGAGCG	21	165	63.0	57.1
	A: GGTGACTGAATTGGCCCTTTCT	22		62.1	50.0
Baat	S: CTTTTCGTGGTGGGAGAAGATG	22	159	61.2	50.0
	A: TGAGCACAGTGGGGAGTAGGGA	22		64.1	59.1
Bcl-2	S: CCGGGAGAACAGGGTATGATAA	22	81		
	A: CCCACTCGTAGCCCCTCTG	19			
Bax	S: AAACTGGTGCTCAAGGCCCT	20	92		
	A: AGCAGCCGCTCACGGAG	17			
Gapdh	S:ATGATTCTACCCACGGCAAG	20	89		
	A:CTGGAAGATGGTGATGGGTT	20			

10	11.59	184.0736	14.1010	20	12.21	802.5603	9.63328	10	9.15	184.0736	12.1067	20	10.14	611.1443	12.1230

Table S6. Primers for qRt-PCR
